# The progress in titanium alloys used as biomedical implants: From the view of reactive oxygen species

**DOI:** 10.3389/fbioe.2022.1092916

**Published:** 2022-12-19

**Authors:** Jun Yang, Chang Liu, Hui Sun, Ying Liu, Zhaogang Liu, Dan Zhang, Gang Zhao, Qiang Wang, Donghong Yang

**Affiliations:** ^1^ School of Stomatology, Jiamusi University, Jiamusi, China; ^2^ Liaoning Provincial Key Laboratory of Oral Diseases, School and Hospital of Stomatology, China Medical University, Shenyang, China; ^3^ The Affiliated Taian City Central Hospital of Qingdao University, Taian, China

**Keywords:** reactive oxygen species, biomaterials, titanium, implants, antibacterial elements, antibacterial coatings

## Abstract

Titanium and Titanium alloys are widely used as biomedical implants in oral and maxillofacial surgery, due to superior mechanical properties and biocompatibility. In specific clinical populations such as the elderly, diabetics and patients with metabolic diseases, the failure rate of medical metal implants is increased significantly, putting them at increased risk of revision surgery. Many studies show that the content of reactive oxygen species (ROS) in the microenvironment of bone tissue surrounding implant materials is increased in patients undergoing revision surgery. In addition, the size and shape of materials, the morphology, wettability, mechanical properties, and other properties play significant roles in the production of ROS. The accumulated ROS break the original balance of oxidation and anti-oxidation, resulting in host oxidative stress. It may accelerate implant degradation mainly by activating inflammatory cells. Peri-implantitis usually leads to a loss of bone mass around the implant, which tends to affect the long-term stability and longevity of implant. Therefore, a great deal of research is urgently needed to focus on developing antibacterial technologies. The addition of active elements to biomedical titanium and titanium alloys greatly reduce the risk of postoperative infection in patients. Besides, innovative technologies are developing new biomaterials surfaces conferring anti-infective properties that rely on the production of ROS. It can be considered that ROS may act as a messenger substance for the communication between the host and the implanted material, which run through the entire wound repair process and play a role that cannot be ignored. It is necessary to understand the interaction between oxidative stress and materials, the effects of oxidative stress products on osseointegration and implant life as well as ROS-induced bactericidal activity. This helps to facilitate the development of a new generation of well-biocompatible implant materials with ROS responsiveness, and ultimately prolong the lifespan of implants.

## 1 Introduction

The oral and maxillofacial region is more susceptible to oxidative stress than other tissues because of its anatomical and physiological position (Wu and Liu, 2019). Dental and maxillofacial bone tissue defect caused by various factors such as periodontal disease, trauma, infection, tumor resection, *etc.* are often achieved by dental implants. However, medical implant materials often face the risk of secondary revision surgery. Not only increases the economic burden of the patient but also causes the patient to suffer unnecessary secondary suffering. Firstly, when the implant is used for bone repair, it is easy to cause bacterial adhesion and accumulation, resulting in postoperative infection (Yuan et al., 2021). Postoperative bacterial infection directly leads to the failure of implant surgery, which is not conducive to the long-term use of implants. Secondly, large number of recent studies showed that the failure of medical implant materials is related to oxidative stress, which occurs when the production of oxidants exceeds the antioxidant capacity of cells or tissues (Yuan et al., 2021). Common oxidants in cells mainly include reactive oxygen species (ROS), nitrogen (RNS), and the resulting lipid peroxidation (LPO) products. ROS act as a kind of signal molecule to maintain life processes such as cell proliferation and differentiation at physiological concentrations (Sies and Jones, 2020). The surface topography, mechanical properties, and degradation products of implant materials all affect ROS production. When the implant used for bone repair, bacterial adhesion and proliferation are easily induced, leading to postoperative infection, which directly cause the failure of implant surgery and is not conducive to the long-term use of implants. To solve this problem, antimicrobial drugs have been used. Traditional treatment usually needs long period of time, which in turn leads to the emergence of drug-resistant bacteria. Therefore, it is important to develop a new and efficient antibacterial method. ROS has a rapid and efficient bactericidal ability, which can effectively destroy DNA, protein and cell membrane without producing drug-resistant bacteria (Xie et al., 2018). The proliferation and adhesion of microorganisms on the surface of the implant can be effectively controlled by changing the elemental composition and surface characteristics of the implant to control the generation of ROS. Therefore, a better understanding of the interaction between medical materials and ROS, as well as ROS-mediated antibacterial effect will provide reference for further research directions and innovative ideas.

## 2 Oxidative stress

### 2.1 The generation and removal of reactive oxygen species

ROS derived from molecular oxygen is a collective term for many related molecules which are formed by redox reactions or electronic excitation (Hawkins and Davies, 2019). ROS can be divided into non-radical and free radicals by the number of free electrons. Among them, two-electron (non-radical) ROS include hydrogen peroxide (H_2_O_2_), Organic hydroperoxides (ROOH), singlet molecular oxygen (^1^O_2_), Ozone (O_3_) Hypochlorous acid and hypobromous acid (HOCl and HOBr) and so on. Free radical ROS include superoxide anion radical (O_2_^−^), Hydroxyl radical (·OH), Peroxyl radical (ROO·) and alkoxyl radical (RO·) (Fernandes et al., 2015; Jones and Sies, 2015; Winterbourn et al., 2016; Galaris et al., 2019; Mascio et al., 2019; Gulcin, 2020). One of the typical oxidants, H_2_O_2_, can regulate cell proliferation, differentiation, migration and angiogenesis through related redox signaling at intracellular physiological concentrations (Saba et al., 2018). However, H_2_O_2_ exceeding the physiological concentration level can lead to non-specific oxidation of proteins and damage to biomolecules such as DNA, RNA, polyunsaturated fatty acids *etc.* and affect their ability to perform their functions (Kreuz and Fischle, 2016). Thus, on the one hand, oxidants act as a switch for molecular redox in signal transduction processes (Bleier et al., 2015). On the other hand, if the accumulated oxidant cannot be removed in time, it will affect the normal life activities of the organism (Lambeth, 2007). However, in organisms, both higher and lower, various organisms evolve a relatively well-established system for scavenging ROS from the body (He et al., 2017). The intracellular mechanism that scavenges ROS and protects cells from oxidative stress damage is called the antioxidant system. In the human body, it is usually achieved through various enzymatic reactions. Antioxidant systems include superoxide dismutase (SOD), catalase (CAT), glutathione peroxidase (GSH-Px) and other enzymatic antioxidants and non-enzymatic antioxidants (He et al., 2017). It is because of the antioxidant system that redox in the cells can be balanced, ROS can be cleared and cells are protected from oxidative damage. For example, H_2_O_2_ can be controlled by sinks and redox relays (Sies and Jones, 2020). Common enzymes such as peroxiredoxins and glutathione peroxidases can remove H_2_O_2_ by catalysis (Chance et al., 1979). In addition, mitochondrial nicotinamide nucleotide transhydrogenase (NNT) is able to scavenge cellular H_2_O_2_ by shifting reducing equivalents from NADH to NADPH (Hanschmann et al., 2013).

### 2.2 Reactive oxygen species and cell function

As mentioned above, intracellular ROS is essential for cell signaling at the physiological level. ROS have been involved in the activation of a variety of cellular signaling pathways and transcription factors, such as mitogen-activated protein kinase (MAPK), phosphoinositide 3-kinase (PI3K)/Akt, nuclear factor (erythroid-derived 2) like protein 2 (Nrf2)/Kelch-like-ECH-associated protein 1 (Keap1), nuclear factor-κB (NF-κB), and tumor suppressor p53 (Bae et al., 2011; Kaminskyy and Zhivotovsky, 2014; Zhang J. et al., 2016). ROS regulate that normal physiological function of cells through the transcription factors, including cell proliferation, differentiation, motility, and cell survival. Luo et al. (2020) incubated bone marrow mesenchymal stem cells (BMMSCs) in osteogenic differentiation medium and detected the ROS increased. However, the up-regulation of ROS significantly alleviated in the co-cultured system of BMMSCs and macrophages. It indicated that macrophages regulate BMMSCs osteogenic differentiation by reducing intracellular oxidative stress (Luo et al., 2020). In addition, it is well-established that ROS play an important role in cell migration by regulating focal adhesions and the cytoskeleton. In the study of Xu, Cobalt-induced ROS inactivate downstream RhoA, impairing the macrophage cytoskeletal reorganization and the cell migration, which could result in a prolonged immune cell retention thereby propagating the chronic inflammation (Xu et al., 2018). A variety of different sources for ROS have been proposed, whereas different sources of ROS also have different effects on cells. NOX4-derived ROS plays an important physiological role in regulating monocyte migration and macrophage recruitment (Lee et al., 2013). Ehnert et al. (2018) verified that monocyte- and macrophage-like cells induce the migration of primary human osteoblasts (phOBs) in a TGF-β-dependent manner. The activation of FAK caused by TGF-β-dependent induction of NOX4 and related production of ROS are the key mediators. It is likely that the induction of autophagy occurs upon exposure to the moderate dose of ROS (Ehnert et al., 2018). This is generally considered a cell survival response, although it can lead to cell death. At sufficient doses, ROS cause severe damage to macromolecules, which can lead to cell death by processes such as apoptosis and/or necrosis (Redza-Dutordoir and Averill-Bates, 2016). Through the pathway of the mitochondrial, ROS also can induce cytochrome c released from mitochondria and induction of apoptosis (Redza-Dutordoir and Averill-Bates, 2016). Besides, Fas-mediated apoptosis can also be activated by ROS such as H_2_O_2_. For implants, Yang F. et al. (2019) confirmed that metallic wear debris-induced ROS is related to the apoptosis of osteoblast. They found that the production of ROS increased, the mitochondrial membrane potential collapsed, and activation of mitochondria-caspase-dependent and endoplasmic reticulum (ER) stress apoptotic pathways in rat primary osteoblasts (Yang F. et al., 2019).

### 2.3 Sources of oxidative stress in the oral cavity

As described by Żukowski et al. (2018) they introduced ozone, ultrasound, low temperature plasma, laser, ultraviolet radiation, fluoride, composite resin, adhesive, orthodontic bracket and wire, and implant materials for oral treatment induced the production of ROS. This suggests that the generation of ROS appears to be unavoidable in any dental treatment. It is reported that more than 50% of the global population is affected by gingivitis and periodontitis (Kumar, 2019), and the degree of oral inflammatory response is positively correlated with the content of ROS and oxidative damage products in oral tissues, gingival crevicular fluid and saliva (Tóthová and Celec, 2017). When the body faces the invasion of microorganisms, the microorganisms induce the activation of phagocytic cells such as granulocytes, monocytes, and macrophages, resulting in the generation of ROS (Moghadam et al., 2021). In fact, almost all inflammatory diseases lead to oxidative stress, and periodontitis, the most common oral disease, as a chronic inflammatory disease also illustrates this point (Sczepanik et al., 2020). In the oral, increased oxidative stress in turn triggers more tissue damage, such as gum tissue and bone tissue, which further exacerbates the development of inflammation. Obviously, these poor oral environment conditions for patients are unfavorable for their health.

### 2.4 Oral diseases and oxidative stress

The pathogenesis and development of oral diseases, such as gingivitis, periodontitis and peri-implant inflammation, are related to oxidative stress. In addition, oral mucosal diseases such as oral lichen planus (OLP) is also related to ROS (Sardaro et al., 2019). More seriously, many suggest ROS participate in the pathogenesis of head and neck cancers (Kesarwala et al., 2016). ROS are involved in the initiation, promotion, and progression of head and neck cancers as described by the three major steps in the classic model of carcinogenesis (Hennings et al., 1993; Goetz and Luch, 2008; Kesarwala et al., 2016). In this review, we introduce the relationship between oral implants and ROS production, and mainly focus on the relationship between ROS and peri-implant inflammation, the long-term stability of implants in the following section.

For peri-implant inflammation, large numbers of studies showed increased ROS expression and ROS relative genes expression in the peri-implant tissue of peri-implantitis patients (Chen M. et al., 2019). Guo et al. (2015) also found that patients with periodontal diseases or peri-implantitis had significantly increased levels of ROS and advanced glycosylation end products (AGEs), which correlated with each other. On the other hand, part of the research focus on the relationship between metal ions or particles in surrounding tissues and inflammation. Souza et al. (2020) studied the effect of titanium particles and ions on biofilm and cell morphology. Their results suggest that titanium particles have the potential to alter the microbial composition of the bacteria biofilms on the surface of titanium, and that the presence of peri-implant titanium products may lead to dysbiosis of peri-implant microorganisms and the development of implant inflammation (Souza et al., 2020). Overall, these results demonstrate that the implant influences the production of ROS and may leads to the progression of inflammation.

### 2.5 Oral disease and long-term stability of implants

Common complications after implant placement are peri-implant mucositis and peri-implantitis. The former is defined as a reversible inflammatory lesion of the mucosa near the surface of the implant abutment (Heitz-Mayfield and Salvi, 2018), while the latter acts as inflammatory process affecting the peri-implant tissue and causing loss of bone tissue (Schwarz et al., 2018). A systematic report by Derks and Tomasi (2015) based on a large random sample and adequate imaging assessment, showed a weighted mean prevalence of peri-implantitis of 22%. However, in another combined retrospective analysis of 588 patients and 2277 implants over a period of 9 years, Derks et al. (2016) reported peri-implantitis (bone loss >0.5 mm) in 45% of patients; 14.5% of patients had moderate to severe peri-implantitis (bone loss >2 mm). These data indicate that the occurrence of per-implant inflammation generally exists in the process of implant treatment. However, this may be related to the large number of titanium particles found widely at the implant site. As described in some articles, titanium particles can be generated and released into peri-implant tissues in a variety of ways, such as electrochemical corrosion, fretting wear, which lead to the occurrence of peri-implant inflammation and even systemic diseases. In the experiment by Soler et al. (2020) they analyzed eight cases of failed implants by SEM, finding that titanium particles were present in the soft tissue in five of them. Furthermore, the surface of the failed implant was rougher than that of the normal implant, which might be due to corrosion of the implant surface caused by the wear of the oxide layer during the implantation process. Nevertheless, this is contrary to the view of Sridhar, who believes that an increase in the surface roughness of the implant is caused by the increase of metal elements and debris between the implant and the surrounding (Sridhar et al., 2016).

In order to explore the relationship between peri-implantitis and ROS, Mijiritsky’s research showed that high levels of oxidative stress-related genes in the inflammatory state inhibited the activation of genes associated with the conversion of MSCs to osteoblasts. And they demonstrated that bone loss in an inflammatory environment may be closely associated with high levels of ROS (Mijiritsky et al., 2019). In a study on the effects of titanium nanoparticles (NPs) on chronic inflammation and osteolysis, Bressan et al. (2019) showed that titanium nanoparticles led to a decrease in the activity of MSCs and fibroblasts and an increase in the production of ROS. Elevated ROS recruited abnormal numbers of neutrophils, produced large amounts of metalloproteinases, and led to the degradation of collagen, which histologically manifested as activation of the osteolytic process (Bressan et al., 2019). The above results indicate that peri-implantitis induces the generation of ROS, and high levels of ROS tend to increase the probability of bone loss and osteolysis, affecting the performance and stability of implants.

## 3 Interaction between implant material factors and reactive oxygen species

Bone is a metabolically active structure because osteoclastic bone resorption and osteoblastic bone formation occur during the entire life of the bone tissue (Boyce et al., 2012). Bone health and homeostasis are the result of a delicate balance between the activity of osteoblasts and osteoclasts. When maxillofacial bone suffers bone defect in response to periodontitis, trauma or tumor, cells such as osteocytes, osteoblasts and osteoclasts will are exposed to oxidative stress (Sheppard et al., 2022). However, ROS may impede or undermine the complicated bone regeneration process through a variety of signaling pathways.

### 3.1 Controlling oxidative stress by implant design

**FIGURE 1:**
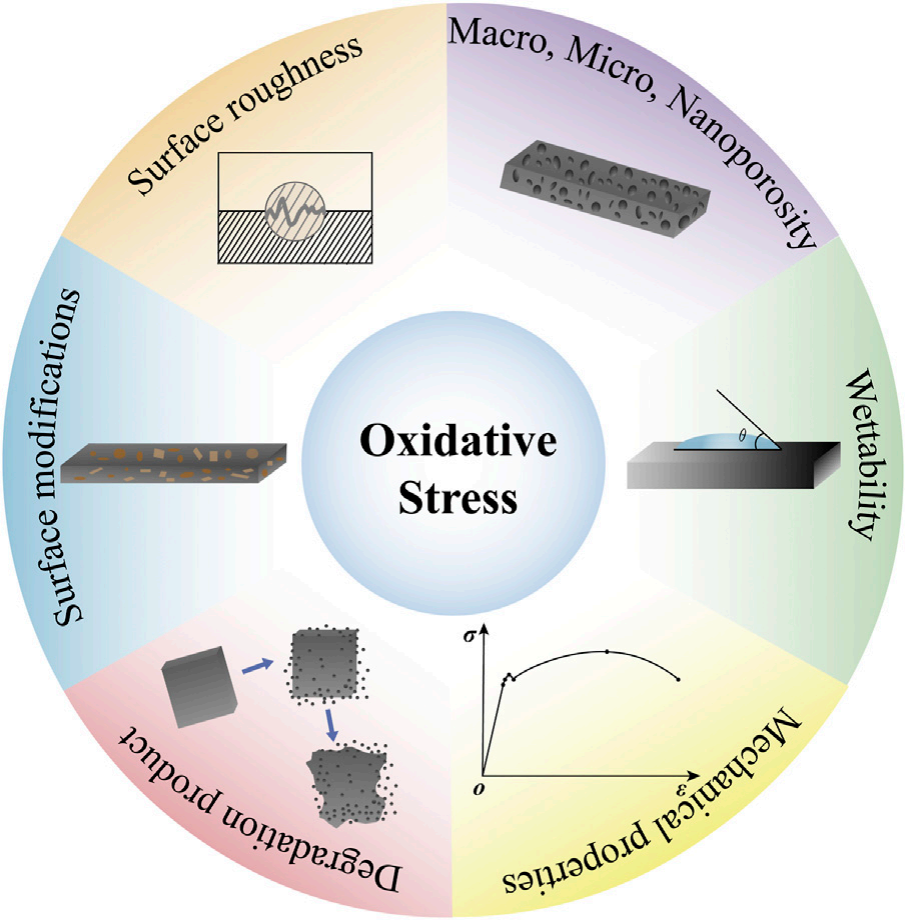
Material factors affecting oxidative stress. Materials factors such as, the size and shape of materials, the morphology, wettability, mechanical properties, and other properties play a significant role in oxidative stress.

In fact, not only in the process of bone regeneration, but also in the early stage of wound healing, both neutrophils and macrophages are affected by different material surfaces like titanium or zirconia. And they will be modulated by surface topography, wettability and stiffness (Nadzam et al., 2000; Ley, 2017; Abaricia et al., 2020). El Kholy et al. (2020) showed that neutrophils reduce respiratory burst and exhibit intact cellular morphology on moderately rough hydrophobic titanium surfaces. Indeed, tissue repair and regeneration are mainly determined by the physical and chemical properties of implants which are tightly linked to the generation of ROS. And biological degradation of medical implant materials (polymers, metals, bioactive glass, *etc.*) occurs over time in the human body. As a result, ions, molecules, and particle fragments of all sizes are produced. Depending on their composition, size, toxicity and release mode, these products will affect the levels of local oxidative stress. Furthermore, the size and shape of materials, the morphology, wettability, mechanical properties, and other properties play a significant role in oxidative stress (Mouthuy et al., 2016). And Table 1 and Figure 1 show these materials factors which can influence the production of ROS.

**TABLE 1:** Material factors affecting oxidative stress.

Factor	Material	Pathways of influence	References
The topography of the material surface	Ti	The multiscale-ordered structure created by the acid-base heat treatment of the surface regulated the production of ROS.	Lao et al. (2020)
Wettability	Ti	After alkali heat treatment, high-energy [001] crystalline facets of anatase phase nanoparticles formed on the Ti surface, promoting water dissociation, and leading to the formation of hydroxyl groups	Hu et al. (2014)
The mechanical properties of materials	Hydrogel	Compared with traditional fibronectin (FN) coating hydrogel with GPa stiffness, polyacrylamide (PAAm) hydrogel with kPa stiffness can make MSCs express more ROS.	Yang et al. (2016)
Size of the degradation products of the material	TiO_2_ particles	TiO_2_ micro-particles induced more ROS production than TiO_2_ nano-particles	Kheder et al. (2021)
The difference of metal ions	Co	Induced oxygen radical production in the form of Co^2+^, and high Co^2+^ concentration induced an increase in HIF-1α expression which led to increased ROS production	Samelko et al. (2013); Chamaon et al. (2019)
	Mg	When magnesium degrading particles was ingested by macrophages, an increase in ROS production is induced	Jin et al. (2020)
	Ti	Titanium ions regulated the key signaling proteins FAK and the balance of phosphorylation of PTP-1B through the oxidative stress pathway, thereby enhancing the adhesion capacity of pre-osteoblasts	Rossi et al. (2017)

#### 3.1.1 The morphology of the material

The topography of the material surface directly determines how cell attach, and ultimately influencing the production of ROS in cells. Due to increased surface area in contact with the surrounding tissues, rough topography is more likely to attract more inflammatory cells like macrophages. As one of the first cells to arrive at the tissue–implant interface, macrophages can mediate early immune response by secreting cytokines, chemokines, and growth factors (Ziats et al., 1988; Gordon et al., 1995). Generally, macrophages prefer rough surfaces to smooth surfaces, a phenomenon called “rugophilia” (Rich and Harris, 1981; Salthouse, 1984). Different roughness of implant surfaces has different effects on macrophage polarization. In the study of Zhang, the smooth surface with the low and medium roughness (i.e., Ra = 0.51–1.36 μm; Sa = 0.66–2.91 μm) induced the polarization of macrophages to M2 phenotype. On the contrary, the high rough surface (Ra = 2.60 μm, Sa = 4.11 μm) can significantly upregulated the secretion of M1-related cytokines, including TNF-α, INDO and CXCL11, and downregulated the secretion of M2-related cytokines, including TGF-β, CCL18, MCR-1 and CCL13 (Zhang Y. et al., 2019). It seems that only a roughness range (Ra = 0.51–1.36 μm, Sa = 0.66–2.91 μm) to polarize the adherent macrophages to M2 phenotype by down-regulating the pro-inflammatory, up-regulating the secretion of anti-inflammatory cytokines, gene expression, and surface markers (Zhang Y. et al., 2019). Similarly, hierarchical micron/submicron topographies with moderate roughness were demonstrated to be beneficial for osseointegration (Wennerberg and Albrektsson, 2010; Medvedev et al., 2016). The macro/micro/nano-rough TiAlV (mmnTiAlV) surface prepared by Olivares-Navarrete et al. (2015) has large pits and craters with superimposed micron- and submicron-scale features. Average roughness (S_a_) for smooth TiAlV (sTiAlV) is 0.27 ± 0.01 μm, and mmnTiAlV is 2.74 ± 0.18 μm mmnTiAlV reduced the local inflammatory environment, decreased levels of pro-inflammatory cytokines, and increased levels of anti-inflammatory cytokine IL-10 compared with the sTiAlV. To sum up, the specific surface roughness is beneficial, not only for macrophage regulation of early inflammation but also for subsequent osseointegration. In addition, the transcription of genes, regulated by rougher surfaces, was confirmed to affect the level of intracellular ROS. Galli et al. (2011) compared C2C12 cells cultured on a smooth titanium surface with a rough titanium surface treated by SLA and found that cells grown on rough surfaces were more resistant to cell damage caused by ROS. And the number of viable cells in the SLA treatment group was higher than that in the polishing treatment group. These are achieved by activating the Wnt/β-catenin signaling pathway and then stimulating the FoxO/β-catenin signaling pathway (Galli et al., 2011). In the study by Lao et al. (2020) they prepared titanium implants with different microstructural features by acid-base heat treatment. The researchers found that the titanium surface structure which had the multiscale-ordered structure could also modulate ROS production and stimulate macrophage M2 polarization to exhibit superior biological capability.

In addition, the dimensionality of nanomaterial was found to correlate with intracellular generation of ROS and cellular mechanical properties, which may interact directly with the filamentous actin cytoskeleton (Pastrana et al., 2019). By comparing two-dimensional layered carbon nanostructures with one-dimensional carbon nanostructure, Pastrana et al. (2019) found that the former one significantly enhanced the adsorption of proteins and reduced the ROS generation. However, the latter one further induced the generation of ROS due to the severe disruption of the cytoskeleton resulting in the reduction of cell elastic modulus. It is seen that when considering the mechanical properties of the materials itself, the impaction of materials on mechanical properties of the cytoskeleton cannot be ignored (Moraes et al., 2011).

#### 3.1.2 Wettability of the material

The physicochemical properties and morphology of the materials determines their wettability, which affects the adsorption of various biomolecules and the attachment of cells (Jurak et al., 2021). These both affect levels of ROS ultimately. Serrano et al. (2005) improved the hydrophilicity of PCL films by alkali treatment, and compared with the untreated films. The treated PCL induced the appearance of oxygen-containing functional groups on the surface of the material and reduced the generation of ROS (Serrano et al., 2005). In addition, the hydrophilic surface reduced bacterial adhesion was achieved by reducing the van der Waals gravitational force between the bacteria and the surface, which is important to the prevention of infection around the implant (Caykara et al., 2020). In a study of Hu et al. (2014) they prepared anatase phase nanostructures on Ti surfaces by alkali heat treatment. They found that the modified functionalized surfaces facilitated the dissociation of water leading to the formation of hydroxyl groups, which formed chemical bonds with undercoordinated Ti atoms and reacted to form ROS (Hu et al., 2014). The hydrophilic anatase has a remarkable antibacterial effect, achieved by the inhibition of bacterial adhesion by surface-generated ROS.

#### 3.1.3 Surface charge on materials

The dynamic cell-material interaction is controlling not only cells survive but also their adhesion and differentiation (Metwally and Stachewicz, 2019). This may be achieved through surface properties such as material surface charge and potential (Kaivosoja et al., 2012; Li et al., 2015). Surface charge determines the amount, type, and extent of folding of the absorbed proteins, which in turn determines the cell adhesion process (Mariani et al., 2019). In addition, different cell species have different responses to surface charge and potential. Interestingly, positively charged surfaces significantly affect cell proliferation and spreading, especially in the early stages of the cellular response. Positive charges activate the signaling cascade of the immune system and the regenerative response to biomaterials (Mariani et al., 2019). The positively charged nanocrystalline surface with higher conductivity are beneficial to MC3T3-E1 cell proliferation (Rahman et al., 2017). Besides, the surface charge can be controlled to reduce bacterial adhesion. In Yang’s study, they fabricated Zr-30Ta and Zr-25Ta-5Ti alloys with negatively charged surfaces, which exhibited better antibacterial properties than CP-Ti. The electrostatic interaction repels the negatively charged bacteria from the alloys, which reduce the bacterial adhesion and prevent biofilm formation. More importantly, Zr-30Ta and Zr-25Ta-5Ti alloys increased ROS production and caused oxidative stress, leading to bacterial death (Yang et al., 2022). The surface charge can render the surface into more hydrophilic, further affects the adhesion and spreading of early cells (Biegaj et al., 2017).

#### 3.1.4 Mechanical properties of the material

The mechanical properties of materials such as elastic modulus, compressive strength and other factors may be another important source of oxidative stress (Nizami et al., 2021). Excellent mechanical properties contribute to the osseointegration of the implant and the immune response of the host. When these mechanical properties do not match those of the host tissue, it may lead to physical damage to the host tissue, which will further lead to the activation of innate immune cells through damage-associated molecular patterns (DAMPs) of cells and extracellular matrix, inducing oxidative stress. More interestingly, compared with traditional fibronectin (FN) coating hydrogel with GPa stiffness, polyacrylamide (PAAm) hydrogel with kPa stiffness can make MSCs express more ROS (Yang et al., 2016). The change of hydrogel stiffness regulates the expression of mechanical sensitive proteins including Rho and Yes-associated-protein (YAP), thus influencing the ROS downstream signaling protein secretion groups behavior, which play critical roles in tissue repairment and cell differentiation (Yang et al., 2016). The decrease of hydrodel stiffness upregulates the ROS expression and affects the HIF1α signaling result in downregulate VEGF-HUVEC angiogenesis (Yang H. et al., 2019). It is also observed that higher matrix stiffness hydrogels induced stronger angiogenesis in HMSC differentiated endothelial cells (Lee et al., 2017).

#### 3.1.5 Degradation products of the material

The generation of ROS and the unsuitable implant materials may exacerbate the degradation, as the result of the trauma which occurs during implantation. Degradation products of different sizes including ions (from metals, ceramics, and polymers), molecular fragments (from polymers) and particulate fragments (from all types of materials) produce different types of biological responses *in vivo*. Ions induce the formation of highly reactive hydroxyl radicals through Fenton reactions, and molecular fragments directly or indirectly induce the formation of oxidants (Mouthuy et al., 2016). However, fragments of macromolecular particles are cleared by inflammatory cells such as macrophages and Foreign Body Giant Cells (FBGCs) *in vivo*, which both release oxidants in the process of clearance. Therefore, regardless of the material, ROS are inevitably generated in the body, affecting the degradation and long-term stability of the implant.

Alloys containing the elements Fe, Co, Cr, Ti, and Ni, as well as newly developed metallic materials containing the elements Cu, Mg, and Ta widely used in clinical practice in recent years (Putra et al., 2020). During the implantation of these metallic materials, the inevitable tissue damage leads to the generation of ROS (Nakkala et al., 2021). In contrast, ROS are more likely to react with the metal material in a redox reaction, producing H_2_O_2_ as an intermediate product and causing electrochemical corrosion. As a result of electrochemical corrosion, high concentrations of metal ions, such as Fe^2+^, Cu^2+^, Cr^6+^, and Co^2+^ are often found at implantation site, and these metal ions promote the formation of hydroxyl radicals through the Fenton reaction under the action of H_2_O_2_ and exist for a long time at implantation site (Di Laura et al., 2017). However, the oral cavity is a complex microenvironment in which intraoral implants are subjected to frequent mechanical loads. On the one hand, this leads to a constant breakdown of the protective oxide layer, which further accelerates wear and electrochemical corrosion. On the other hand, the complex body fluid composition affects the corrosion process of metals, resulting in various forms of degradation products, including free metal ions, colloidal complexes, inorganic metal salts or oxides, organic form, and wear particles (Mouthuy et al., 2016). Recent studies show that different sizes of metal degradation particles have different effects on cells and the production of ROS. Compared with nanoscale TiO_2_ particles, Kheder et al. (2021) found that micron-scale TiO_2_ particles co-cultured with THP-1 monocytes produced higher levels of ROS, and the nanoscale group showed a concentration-dependent increase in ROS production. As can be seen, the production of ROS and cellular biological responses of different metal degradation products are different.

#### 3.1.6 The difference of metal ions

When titanium and its alloys were not widely used in clinical applications, cobalt alloys such as CoCrMo were used extensively in total hip arthroplasty, and then for a long time in orthopedics and dentistry. It has been found through long-term clinical observation that cobalt alloys *in vivo* lead to the formation of cobalt ions and alloy particles due to corrosion, and then exert cytotoxic effects in a dose-dependent manner (Bijukumar et al., 2018). Through *in vitro* clinical data analysis, Samelko et al. (2013) found increased HIF-1α expression in the peri-implant tissues and body fluids of patients in the cobalt alloy implant group and a significant increase in implant failure rates (Samelko et al., 2013). The *in vivo* experiments showed that cobalt alloy particles induced a significant increase in HIF-1α, VEGF, TNF-α and ROS expression in THP-1 cells, whereas titanium alloy particles produced similar effects only at higher excitation doses (Samelko et al., 2013). However, Chamaon et al. (2019) compared the effects of cobalt ions with cobalt alloy particles on cells, finding that it was cobalt ions, rather than the cobalt alloy particles, that led to the formation of oxygen radicals (Chamaon et al., 2019). Cobalt in its ionic form not only induced the formation of free radicals in cells, but also generated cytoplasmic superoxide, which fundamentally induced oxidative stress. Interestingly, no adverse effects of cobalt particles on superoxide anions were observed in their experiments, contrary to the view of Raghunathan et al. (2013).

Titanium and its alloys are the most widely used metals materials for dental implant treatment and orthopedic implants due to their good biocompatibility and excellent mechanical properties. When titanium is exposed to air, a passivated, corrosion-resistant oxide film can be instantly formed on its surface. However, the inert oxide film still undergoes some electrochemical corrosion in the oral environment due to the presence of complex electrolytes such as saliva and blood, leading to the release of titanium ions and titanium dioxide nanoparticles. This usually manifests clinically as soft tissue discolouration and bone resorption (Wachi et al., 2015). Thus, Tsaryk et al. (2007) showed that endothelial cells under oxidative stress had increased lactate dehydrogenase (LDH) release, reduced SOD activity and significantly decreased glutathione (GSH) concentration on the Ti6Al4V surface, leading to an increase in the number of necrotic cells. Kalbacova et al. (2007) found that cathodic polarization of Ti6Al4V induced an increase in intracellular ROS levels in osteoblasts and macrophages, and inhibited cellular metabolic activity in a dose-dependent manner. This result is consistent with that obtained by Tsaryk et al. (2007) above after creating an oxidative stress model by the addition of H_2_O_2_ to cells, and macrophages showed a higher tolerance to oxidative stress than osteoblasts. There are many opinions about the range of concentrations for the different ions which promoting inflammatory reaction. In a previous phase, Taira et al. (2006) explored the effect of Ti concentration on RAW 264.7 cells. RAW 264.7 cells cultured in the medium with 1 ppm Ti for 2-days exhibited cell viability of about 60%, SOD production and TNF-α secretion had increased which relative to those of the control cells cultured in the medium without Ti (Taira et al., 2006). It is speculated that this might be related to oxidative stress and inflammatory response caused by macrophages engulfing titanium-containing complexes. Mine et al. (2010) put forward different opinions. They proposed that less than 9 ppm of Ti ions had little effect on the viabilities of RAW 264.7 cells, whereas 20 ppm of Ti ions significantly decreased the viabilities of them. The difference may be due to the different exposure time of macrophages in the titanium-containing environment. As for GE-1 cells, Makihira et al. (2010) found that cellular responses were induced by Ti ions at less than 9 ppm within 24 h and had little effect on cellular vitality. Ti ions will behave as an inducer of necrosis and be incorporated by GE-1 cells when the concentration of Ti ions is greater than 11 ppm. Furthermore, Titanium ions in concentrations of 9 ppm act synergistically with *Pg*-LPS to increase the expression of CCL2 whereas Ti ions alone elevated ICAM-1 and TLR-4 mRNAs in GE-1 cells (Makihira et al., 2010). Wachi et al. (2015) explored MC3T3-E1 cells and reached similar experimental conclusions. They found that Ti ions may alter osteoclast differentiation by changing the sensitivity of the epithelium that typically surrounds implants to microorganisms (Wachi et al., 2015). Contrary to the view of Wachi et al. (2015); Pettersson et al. (2017) suggest that stimulation of the proinflammatory response is caused by titanium-based particles rather than Ti ions. They explored the effects of different metal ions on THP-1 cells. After 24 h of exposure, neither Co nor Cr had any effect on viability at concentrations of ≥200 μM, and Ti and Mo caused reduced viability. In the mucosal biopsies of three patients, the concentration of titanium-based particles ranged from 7.3 to 38.9 μm *in vivo*. Within these levels, Ti particles could initiate activation and secretion of IL-1b *in vitro* (Pettersson et al., 2017)*.* However, the proinflammatory activity induced by titanium disappeared after filtered (0.22 μm), suggesting that the role of the particles was more important than ions (Pettersson et al., 2017).

The above results showed that titanium ions and their degradation particles could directly induce the occurrence of cellular oxidative stress. However, some results suggest that ROS also act as a signaling molecule in conjunction with titanium ions and their degradation products to affect cell survival through inflammation and other signaling pathways (Li et al., 2020). The experiment by Li et al. (2020) showed that titanium ions promoted ROS production in T cells and led to increased mRNA expression of NLRP3 and CASP1 and secretion of IL-1β, which was reversed by an ROS inhibitor. This indicated that titanium ions activated inflammasome *in vitro* through the oxidative stress pathway and thus induced periodontitis and peri-implantitis (Li et al., 2020). In a study by Rossi et al. (2017) they found that the release of titanium ions from implants regulated the balance of phosphorylation of signaling proteins FAK and PTP-1B through the oxidative stress pathway, thereby enhancing the adhesion ability of pre-osteoblasts.

As the fourth most abundant mineral in the human body, more than half of which is concentrated in bone tissue, magnesium is an important nutrient element for the human body and is involved in a variety of physiological activities (Ciosek et al., 2021). It has attracted widespread attention due to its light weight, good tensile strength, and low elastic modulus close to that of human compact bone (Sheikh et al., 2015). By comparing different concentrations of Ti and Mg particles, Wang N. et al. (2020) found that a specific concentration of Mg particles reversed the reduction in SAOS2 cell viability caused by Ti particles. And Ti particles produced more ROS by disrupting mitochondria, while this was reversed by the addition of Mg particles (Wang N. et al., 2020). However, the results of a recent study showed that degraded particles of magnesium might contribute to the progression of the inflammatory response (Jin et al., 2020). Jin et al. (2020) were the first to explore the potential effects of Mg degrading particles (DPs) on macrophages. Their study showed that DPs from Mg-based alloys were phagocytosed by macrophages and induced overproduction of ROS, which in turn upregulated the expression of several pro-inflammatory cytokines in macrophages, leading to an excessive inflammatory response. These experimental results refine the understanding of magnesium-based materials and will contribute to a better understanding of the health effects of magnesium-based materials when used for tissue repair.

### 3.2 Oxidative stress factors affecting implant materials

**FIGURE 2:**
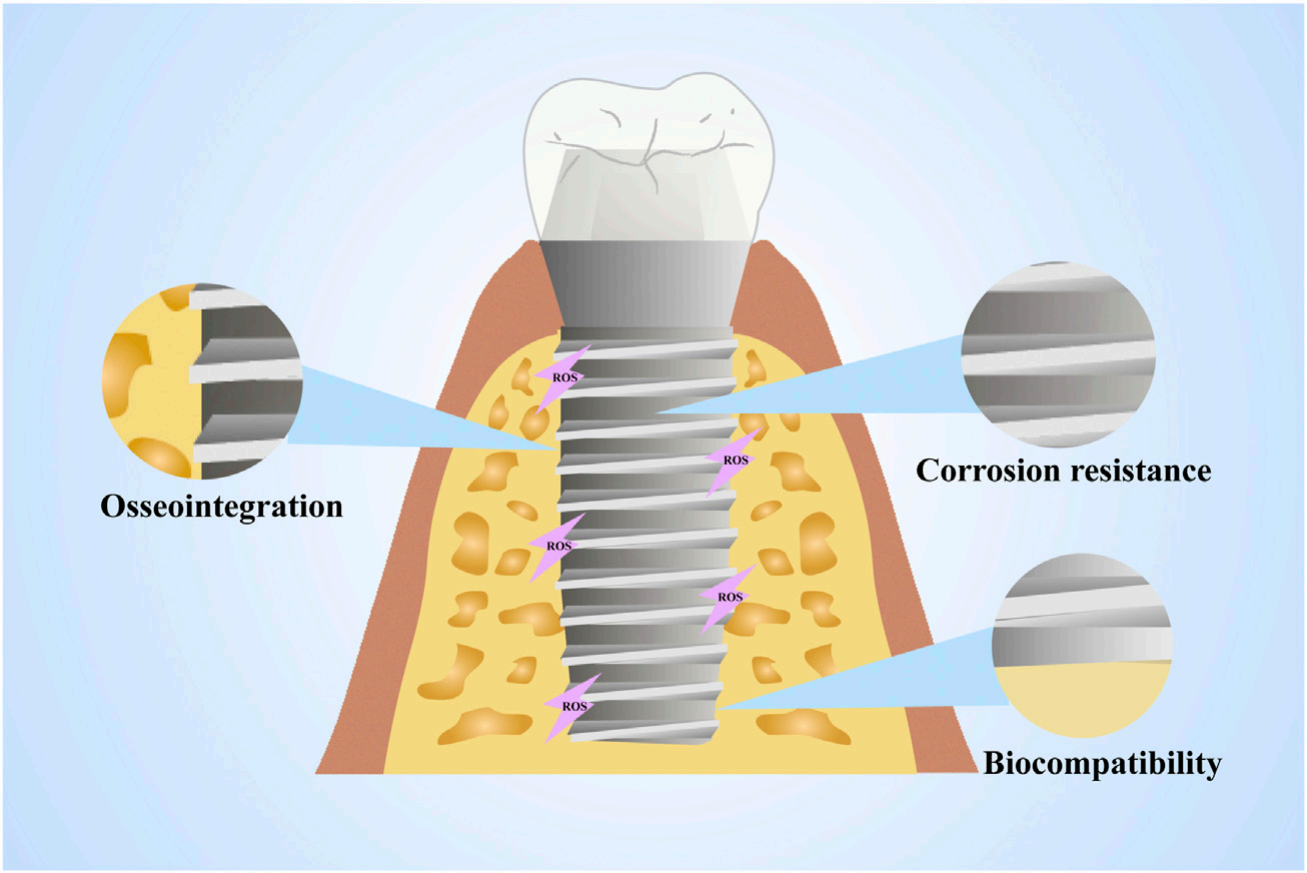
Material properties affected by oxidative stress. The properties of materials such as osseointegration, corrosion resistance and biocompatibility can be affected by oxidative stress.

A wide range of material properties and degradation products of implant materials can influence the occurrence of oxidative stress. However, when considering the properties of a material to induce a biological response, we often consider this to be an inherent characteristic of the material, ignoring the interaction between the material and the host tissue. Indeed, a growing body of clinical data suggests that the host environment responds to materials quite differently. For example, there are dramatic differences in the properties of biomaterials under healthy and diseased conditions (Ratner, 2015). It is thus essential to consider the direct and indirect effects of the host environment on the material while discussing the influence of material properties on oxidative stress. In addition, a kind of factors such as osseointegration, corrosion resistance and biocompatibility can be affected by oxidative stress, as illustrated in Table 2 and Figure 2.

**TABLE 2:** Material properties affected by oxidative stress.

Factor	Affected material properties	Pathways of influence	References
Hyperglycemia	Osteoconductive	The mitochondria and cytosol in a hyperglycemic state produced excessive ROS, which in turn affected the osteoconductive properties of the material and led to implant failure	Yu et al. (2011)
	Osseointegration	The increase in AGEs in peripheral bone tissue in the hyperglycemic state led to an increase in NADPH oxidase in vascular endothelial cells, dysfunction of vascular endothelial cells and impaired angiogenesis at the bone-implant interface (BII)	Hu et al. (2018)
Inflammation	Corrosion resistance	It had been confirmed that the ROS levels was significantly increased in the inflammatory environment, which led to the electrochemical corrosion of inert metal oxide films and consequent degradation of titanium alloys	Fonseca-García et al. (2016)
	Mechanical properties	High-level expression of mitochondrial genes associated with ROS production in the tissues of patients with periodontitis and peri-implantitis affected the long-term stability of the implants	Mijiritsky et al. (2019)
Microorganisms	Corrosion resistance	Microorganisms affected the activation of immune cells and lead to the generation of ROS. Electrochemical corrosion resulted in the production of titanium particles, which could lead to microbial population imbalance and peri-implantitis	Souza et al. (2020)
Fretting friction	Corrosion resistance	The metal shedding caused by fretting friction caused tribocorrosion and generated ROS as an intermediate product, which increased the corrosion rate of the implant	Soler et al. (2020)

#### 3.2.1 The corrosion resistance of implant materials

The damage to the materials’ corrosion resistance and unfavorable consequences brought by ROS became a focus of interest. When the titanium-based material is exposed to oxygen, a passive oxide film forms on the surface in a very short time, making it a biologically inert material. Even if the inert oxide film is destroyed, it quickly forms in the presence of oxygen and protect the titanium surface again (Kim et al., 2019). In the harsh oral environment, this passive oxide film is very stable even if it is subjected to long-term mechanical and chemical stimulation, thereby protecting the titanium surface from corrosion (Gilbert and Kubacki, 2016; Kotsakis et al., 2016). However, it has recently been reported that peri-implantitis still occurs in up to 20% of patients who undergo implantation, resulting in bone tissue loss around the implant. This leads to an ultimate implant failure and removal (Kotsakis et al., 2016).

A recent study showed that Ti corrosion products around implants were greatly increased in patients with peri-implantitis compared to those without peri-implantitis (Mombelli et al., 2018). This result indicates that the microenvironment to which the implant is exposed is highly corrosive during the development of peri-implant inflammation, leading to an attack on the passively generated TiO_2_ film. However, some studies illustrated that the content of ROS in the peri-implant inflammatory microenvironment is significantly increased, and the presence of ROS causes electrochemical corrosion of implant materials under oxidative stress. This is mainly because metal-induced free radical formation alters the corrosion behaviour of metal materials by destroying the protective oxide film that forms spontaneously on the surface of the metal implant, affecting the performance of the metal implant.

#### 3.2.2 The osteoconductive and osseointegration of implant materials

Titanium-based alloys are the most widely used metal implant material in dentistry and orthopedics due to their good mechanical strength and elastic properties. They meet the long-term load in the human body and perform normal physiological functions, which is mostly due to the excellent bone conduction capacity (Li et al., 2020). However, in some systemic disease conditions, its bone conduction capacity might be significantly reduced. Diabetes mellitus, one of the most typical systemic diseases, which adversely affect the bone regeneration and the osteoconductive properties of implants due to the disruption of glucose metabolism in patients (Camargo et al., 2017). Clinical data show that the failure rate of implants in diabetic patients is as high as 10%–20%, which is higher than the normal rate of 1%–3%, making diabetes a typical contraindication for implant treatment nowadays. In recent years, most studies have shown that the failure of implants caused by diabetes is closely related to the excessive production of ROS in intracellular mitochondria and cytoplasm in the hyperglycemic state (Annibali et al., 2016). The excessive production of ROS leads to the occurrence of adverse conditions such as slow bone regeneration and impaired healing after implant placement, which fundamentally allows for a reduction in mechanical retention between the implant and surrounding tissues (Yu et al., 2011). This has a negative impact on the biological properties of the implants. Feng et al. (2013) demonstrated that the excessive production of ROS in the diabetic state led to osteoblast dysfunction and apoptosis, which in turn led to impaired healing of the porous titanium implants to the bone tissue. The cellular stress induced by superoxide anion was attenuated after administration of the antioxidant N-acetylcysteine (NAC), which in turn significantly attenuated the osteogenesis inhibition of porous titanium alloys in diabetic pathological states (Feng et al., 2013). Thereby, early antioxidant treatment and the development of ROS-responsive implants are undoubtedly useful strategies to improve the osseointegration and osteoconductive properties of implants in cases of increased ROS production induced by metabolic disorders *in vivo* (Hu et al., 2018). In addition to early antioxidant treatment, some natural compounds with antioxidant properties including polyphenols, proanthocyanidins (PAC) and silk fibroin are loaded onto the surface of titanium (Tang et al., 2020; Ma et al., 2021; Riccucci et al., 2021). In addition, they can provide a long-term removal of ROS function. Riccucci et al. (2021) loaded polyphenols extracted from organic red grape pomace onto the surface of titanium and exert the effects of polyphenol oxidation resistance and free radical removal, making titanium alloys with anti-inflammatory and antibacterial capability. Moreover, functionalized implant materials by ROS will be discussed in the next section.

## 4 Titanium alloys with antibacterial properties in a reactive oxygen species perspective

Titanium and titanium alloys have been widely used as dental implants and hip prostheses owing to their desirable corrosion resistance and biocompatibility (Saruta et al., 2019). However, microorganisms will attach to the titanium surface to form plaque after implantation leading to inflammation around the implant (Alves et al., 2022). The survival rate of dental implants at 10–15 years is up to 89% plus, but dental implants infections or peri-implantitis may be as high as 14% (Norowski and Bumgardner, 2009). In a retrospective study (Han et al., 2014), further statistics showed that 47% of early implant failures could be attributed to inflammation. This indicates that implant-related infections caused by biofilm on the surface of biomaterials are a serious problem in clinic. Generally, infection begins when bacteria attach to the surface of implants, after the beginning of colonization, with colonization increase then biofilm formed (Jiao et al., 2021). The extracellular polymeric substance (EPS) matrix in biofilm which enable to recalcitrant to host immunity (Hahn and Gunn, 2020) and inhibits antimicrobial agent diffusion (Wang J. et al., 2020). Although pure titanium has excellent mechanical properties, its biological inertness (Kaur and Singh, 2019) and absence of antibacterial properties prevent the application of pure titanium in biomedicine. Accordingly, improving antibacterial properties has become an important direction of current research. Currently, ROS-mediated antibacterial effects can be produced by improving antibacterial properties, mainly through two approaches: changing the elemental composition and surface modifications.

### 4.1 Controlling oxidative stress by introducing elements

At present, antibacterial properties of titanium alloys mainly by adding inorganic antibacterial agents, such as copper (Cu) and silver (Ag), their broad-spectrum antibacterial activity is reported in relative literatures (Ferraris and Spriano, 2016; Wang Y. et al., 2020; Zhang E. et al., 2021). Copper is an essential trace element in human body and involves in a variety of physiological mechanisms (Jacobs et al., 2020). Silver has been used in ancient times (Ahmed et al., 2016) because of its excellent antibacterial capability. In the 19th century, after people realized that microbes could cause infection, silver was used as an antimicrobial in medicine (Talapko et al., 2020). Medical biomaterials containing copper and silver also exhibit anti-attachment and anti-biofilm properties (Zheng et al., 2011; Liu et al., 2016; Lei et al., 2018). The addition of antibacterial elements can make titanium alloys resist infection and inflammation because of antimicrobial elements can promote the production of ROS as the main antimicrobial substance under some conditions, which reacts with cellular metabolites, genetic material, and enzyme to generate oxidative stress. The role of ROS in Cu-bearing and Ag-bearing titanium alloys with antibacterial properties are listed in Table 3.

**TABLE 3:** Controlling oxidative stress by introducing antibacterial elements.

Sample	Manufacture method	Test strains	Antibacterial mechanism	References
Ti-Cu	As cast	*S. aureus*	Ti-Cu alloy can induce ROS generation whether by UMAO treatment or not. In UMAO-treated Ti-Cu alloys, the formation of the CuO phase triggers the production of superoxide by bacterial cells, which leads to the decrease of bacterial metabolic activity	Hu et al. (2020)
Ti6Al4V5Cu	Anneal	*S. aureus*	Cu ions destroyed the activity of the respiratory chain and then produce a large amount of ROS, leading to the membrane disruption. The released Cu ions clearly interfered with the gene replication process	Li et al. (2016)
Ti6Al4V5Cu	Water-cooled induction skull melting	*S. aureus*	Ti_2_Cu are extensively synthesized in the Ti matrix. The Ti matrix and Ti_2_Cu may form a micro-galvanic cell and then initiate electron transfer between the alloy and Cu-rich precipitate, which product ROS.	Xu et al. (2021b)
		*E. coli*		
Ti-Ag	As cast/water quench/precipitate	*S. aureus*	The presence of nanoscale Ti_2_Ag caused high expression of ROS.	Fu et al. (2021)
Ti-Ag	Spark plasma system (SPS)	*S. aureus*	Ag-rich phases induce a high expression of ROS. ROS promoted by Ti_2_Ag and Ag-rich phases disrupts bacterial homeostasis and resulting the bacteria death	Fu et al. (2021)

#### 4.1.1 Copper-bearing titanium alloys

Up to now, there are two viewpoints about the antibacterial mechanism of Cu-bearing titanium alloys: Cu ions release (Chaturvedi and Henderson, 2014) and contact killing (Xu et al., 2021a). Among them, the antibacterial mechanisms of Cu ions include membrane damage (Karlsson et al., 2013), protein denaturation (Vincent et al., 2018) and oxidative stress (Chaturvedi and Henderson, 2014).

Many studies have attributed the antibacterial activity of Cu to ROS-induced oxidative stress under aerobic conditions, mainly initiated by Cu^2+^ that released (Vincent et al., 2018). According to the classical view, redox potential enables Cu to produce hydroxyl radicals *via* Fenton reactions (Chaturvedi and Henderson, 2014; Salah et al., 2021). ·O_2_^−^, NADPH oxidase from the respiratory and intracellular thiols can reduce Cu^2+^ to Cu^1+^ (Dalecki et al., 2017). Cu^1+^ can react with H_2_O_2_ to Cu^2+^ in OH and continue to produce OH (Godoy-Gallardo et al., 2021). These harmful hydroxyl radicals may lead to membrane rupture because of lipid peroxidation damage and decreasing membrane fluidity (Godoy-Gallardo et al., 2021). Li et al. (2016) support this view and they proposed that the Cu ions released from the alloys, which destroy the permeability of the bacterial membrane, leading to leakage of reducing sugar and protein from cells. In this study, DCFH-DA probe was used by authors to evaluate the ROS concentration. Compared with the blank control group, the fluorescence intensity produced as an indicator of ROS formation in Ti6Al4V5Cu group increased significantly with longer exposure, which clarify the formation of ROS. In addition, ROS causes lipid peroxidation of the cell membrane by reducing its integrity and fluidity, and then leading to membrane disruption. DNA is also main target of ROS. The released Cu ions showed obvious genotoxicity, owing to interfere the replication of *nuc* and 16SrRNA genes.

In recent years, with the in-depth study, the antibacterial mechanism of Cu-bearing titanium alloys is controversial. Some scholars believe that the antibacterial activity is due to the release of Cu ions, while some scholars propose that it is related to the precipitation phase such as Ti_2_Cu in the alloys (Zhang E. et al., 2016; Peng et al., 2018; Xu et al., 2021a). Xu et al. (2021a) found that the outstanding antibacterial ability against *Staphylococcus aureus* (*S. aureus*) and *Escherichia coli* (*E. coli*) of Ti6Al4V5Cu comes from contact killing *via* the extensive precipitation of Ti_2_Cu instead of Cu ions. Although production of ROS was not evaluated in this study, according to the previous study (Zhang Z. et al., 2019), the precipitated Ti_2_Cu might form micro-galvanic cells with Ti matrix and the electrons transfer between the Cu-rich precipitate and the alloys. Under such conditions, proton motive force is destroyed, inhibit the ATP formation, and accelerate the intracellular ROS production (Zhang Z. et al., 2019).

#### 4.1.2 Silver-bearing titanium alloys

Although Ag has been extensively studied, the exact mechanism of its antibacterial effect is not clear. Currently, the most commonly proposed antibacterial mechanism for silver compounds (Kedziora et al., 2018): extensive disruption of cellular functions due to direct damage to cell membranes or intracellular biomolecules, as well as induction of oxidative stress through ROS production, culminating in the formation of free radicals and extensive cellular damage (Chernousova and Epple, 2013).

It is generally believed that Ag-containing particles in Ti-Ag alloy mainly control the final antibacterial activity, while the release of Ag ions also contributes to the improvement of antibacterial properties (Zhang E. et al., 2021). In Shi’s study, they propose that Ti_2_Ag phase played a major role in the antibacterial properties of Ti-Ag alloy (Shi et al., 2020). An *in vitro* experiment of Ti-Ag sintered alloys (Chen et al., 2016) illustrates similar conclusion. Authors show that to obtain stable antibacterial activity against *S. aureus* bacteria attribute to the formation and distribution of Ti_2_Ag phase. Besides, the Ag content has to be at least 3 wt%. With the increase of Ag content, the antibacterial effect of Ti-Ag alloy is enhanced.

Therefore, Fu et al. (2021) design Ti-Ag alloy with different existing forms of Ag in their study. They observed the overproduction of ROS in Ti-Ag sample with Ag-containing phase by DCFH-DA fluorescence imaging. The Ag-containing phase group also had higher intracellular superoxide levels and protein leakage than the other groups. Meanwhile, cell membranes broken were found on Ti-Ag samples with Ag-containing phase. It means that Ag-containing phase, as the main control factor of the antibacterial properties of Ti-Ag alloy, induces oxidative stress within the bacteria and plays a key role in killing the bacteria.

To sum up, it can be concluded that, the antibacterial behavior of Ti-Ag alloy is that the production of ROS increases the permeability of cell membrane, destroys the integrity of the cell membranes, causes protein leakage and results in the destruction of bacterial cells, finally leads to cell death. In addition, they propose that ROS generated through the electron transfer between Ag-rich phase and Ti_2_Ag when self-corrosion occurs (Fu et al., 2021).

### 4.2 Controlling oxidative stress by surface modification

The addition of antibacterial elements such as Cu and Ag may alter the microstructure characteristics of titanium alloys, thereby potentially altering the mechanical properties and corrosion resistance (Zhang E. et al., 2021). To avoid this problem, researchers synthesize implant surfaces with biocompatible and excellent antibacterial properties by surface modification. We sum up different representative strategies to prevent infection are reported onto titanium and titanium alloy surfaces such as nanoparticles coatings, photosensitive antibacterial surfaces and plasma treatment surfaces in which ROS play a significant role (Table 4).

**TABLE 4:** Controlling oxidative stress by surface modification.

Substrate material	Coating material	Test stains	Antibacterial mechanism	References
**Ti**	TiO_2_ nanopillars	*S. aureus*	The nanopillars can deform and even penetrate the bacterial envelope without causing mechanical rupture. Nanopillars can induce oxidative stress upon contact with bacterial cells, and the cumulative effect of oxidative stress may impair processes such as bacterial growth and biofilm growth and biofilm formation	Jenkins et al. (2020)
		*E. coli*		
		*Klebsiella pneumoniae*		
**Ti**	ZnO nanorod-arrayed coatings	*S. aureus*	Mechanical permeability and ROS were the main factors inhibiting bacterial adhesion. The influx of extracellular H_2_O_2_ and Zn^2+^ into the cytoplasm leads to an increase in intracellular ROS. The quantitative contribution of ROS to antibacterial activity was about 20% during the initial incubation period	Ye et al. (2022)
		*E. coli*		
**Ti**	Ag-NPs	*S. aureus*	The electron transport between Ag-NPs and Ti substrate produced a large amount of ROS in bacterial cells and culture medium. ROS-induced physiological changes, such as intracellular oxidation protein release, and membrane potential reduction, are crucial to bacterial death	Wang et al. (2017)
		*E. coli*		
**Ti**	TNS	*S. aureus*	UV irradiation improved the antibacterial properties of TNS and reduced the viability of *S. aureus* on the material surface by producing ROS.	Hatoko et al. (2019)
**Ti**	Chitosan/Ag/MoS_2_	*S. aureus*	Photoexcited electrons are rapidly transferred from MoS_2_ to Ag-NPs, resulting in higher generation of ROS to kill bacteria. The coverage of CS gives the composite coating a positive charge, which further improves the antibacterial properties of the coating. In addition, CS/Ag/MoS_2_-Ti also showed some photothermal effects	Zhu et al. (2020)
		*E. coli*		
**Ti**	Zn-doped TiO_2_ coating	*S. aureus*	The voltage of PEO treatment affects the ROS release. The weaken of Zn-O bonds in amorphism by PEO treatment product the ROS and promote the Zn^2+^ on the surface of materials, which induced the intracellular ROS production, leading to the bacterial death	Ye et al. (2020)
		*E. coli*		

#### 4.2.1 Nanoparticles coatings

Nanoparticles are a type of particles, which dimensions<100 nm (Yu et al., 2020). Different physical and chemical properties endow NPs with different functions depending on their shape and size (Yu et al., 2020). Different coating techniques such as electron beam evaporation, anodization, plasma spraying *etc.,* can be used to synthesize nanoparticles coatings (Pugazhendhi et al., 2021). Currently, nanoparticles coatings are generally considered to have antibacterial properties, and a growing body of research is under way to induce bacterial death by external production of ROS.

**FIGURE 3:**
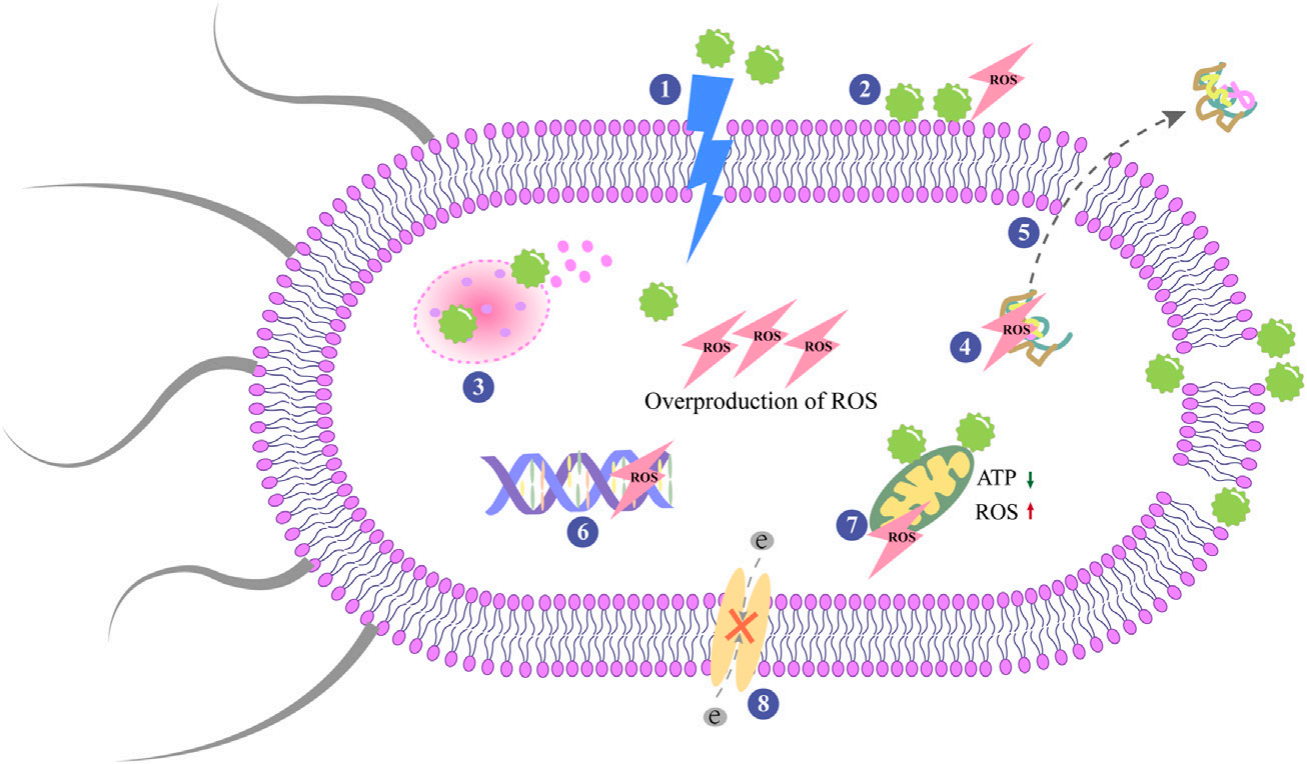
Antibacterial mechanisms on nanoparticles surfaces. The central modes of actions are: 1) The rupture and perforation of membrane; 2) ROS production; 3) Lysosomal destroyed by NPs and result in the release of the contents; 4) Protein damage; 5) Protein leakage; 6) Inhibition of DNA replication; 7) Mitochondrial dysfunctioning; 8) Interruption of electron transportation.

Antibacterial activity of NPs often going along two main interconnected pathways, in many cases simultaneously:1) physical route *via* disruption of membrane potential and integrity and 2) chemical routes *via* the production of ROS (Bhattacharya and Neogi, 2019). When NPs are attached to the bacterial membrane by electrostatic interactions, Vander Waals forces, receptor-ligand, or hydrophobic interactions, and the membrane morphology changes (Shaikh et al., 2019). Extracellular metal nanomaterials have the ability to provide electrons to molecular oxygen, causing the free radical reaction to produce ROS (Godoy-Gallardo et al., 2021). Molecular oxygen is reduced to water through a series of proton-electron transfer reactions, and ATP is synthesized (Bhattacharya and Neogi, 2019). In this process, oxygen is not completely reduced forms superoxide anion and other oxygen-containing radicals (Bhattacharya and Neogi, 2019). Figure 3 depicts the antibacterial process of the nanomaterial surfaces. It should be noted that the process presented in the figure is not exclusive, as antibacterial activity is the complex result of multiple and often interrelated mechanisms.

Jenkins et al. (2020) proposed that TiO_2_ nanopillars prepared on the Ti surface induced envelope deformation and penetration and there was no evidence to support that mechanical rupture and bacterial lysis occurred. This suggests that the bactericidal activity of the nanopillars is not entirely dependent on contact killing and it is mostly link to oxidative stress. When nanopillars contacts with the bacterial cell membrane, it increases oxidative stress proteins such as superoxide dismutase and increases the level of H_2_O_2_. The increased quantity of H_2_O_2_ induce changes in envelope morphology. Nanopillar can also enhance anti-biofilm properties by reducing the ability of bacteria to replicate on the surface by inducing cell impedance.

Ye et al. (2022) conducted in-depth study and the antibacterial activity of ZnO nanorod-arrayed coatings (ZNR) on Ti was quantified by determining the contribution of ROS. For the adhered bacteria, they showed that ZNR-induced ROS (O_2_^−^ and HO·) as well as H_2_O_2_ played a key role in killing *S. aureus* during the initial incubation period, contributing approximately 20%. The extracellular H_2_O_2_ and Zn^2+^ into the cytoplasm leads to the increase of intracellular ROS not only aggravates the phospholipid and plasma membrane damage caused by extracellular ROS, but also causes additional damage to intracellular proteins and DNA, and finally accelerates the death of bacteria. After 2 h of incubation, mechanical penetration becomes the main contributing factor of its bactericidal activity, with a contribution rate of about 96%.

The physical properties of nano-scale have an important impact on the antibacterial behavior (Zhang X. et al., 2021). Generally speaking, smaller size NPs have high antibacterial activity due to their relatively large surface-to-volume ratio, thereby increasing their ability to produce ROS, which in turn destroy bacterial biomolecules, protein, and lipids (Shaikh et al., 2019).

The introduction of Ag-NPs and Cu-NPs on the surface of Ti-based materials can also obtain good antibacterial activity. The antimicrobial activity of Ag-NPs is derived from the alteration of cell membrane integrity and permeability due to ROS production, as well as the release of cellular contents (Dasgupta and Ramalingam, 2016). The study of Wang et al. (2017) provides support for this view. By comparing the antibacterial behavior of Ag-NPs@Ti with and without the addition of the antioxidant N-acetylcysteine (NAC), they found that the antibacterial capacity was inhibited after the addition of NAC, suggesting that ROS was the key reason of the antibacterial effect. Physiological changes induced by ROS, such as intracellular oxidation, protein release, and membrane potential reduction, are crucial to bacterial death. During solution immersion, embedded Ag-NPs in Ti enhanced electron transport, and the micro-galvanic couples between Ti substrate and Ag-NPs can be easily activated. By going through Ag-NPs, electrons by Ti can reach bacteria’s intracellular part, disrupt electron transport, and cause a higher level of intracellular ROS production. Liu et al. (2021) developed the nanoparticles coating on Ti-35Nb-2Ta-3Zr substrate, the surface was structured with titanium nanotubes (NT) loaded with Ag-NPs. Ag-NPs modifications inhibited the growth of *S. aureus* and *E. coli*. It can be considered that the antibacterial effect of Ag-NPs in the current research is relatively clear, and the challenging key point is the to find the balance between the antibacterial activity and cytotoxicity of Ag-NPs. To solve this problem, Cazzola et al. (2022) conduct a study by changing different parameters to tailor the amount of silver and its distribution across the surface oxide layer thickness on Ti6Al4V surface. The samples prepared with the higher concentration of silver precursor can active excellent antibacterial properties. Unfortunately, these samples perform highly cytotoxic. Therefore, they deduced that the silver amount may not the only factor influencing the biological properties. Yang et al. (2018) put forward different viewpoint for this problem. They embedded Ag-NPs into a Ti6Al4V substrate by friction stir processing (FSP). Ag-NPs were placed in preformed troughs, and the distribution of Ag-NPs is related to the depth of the preformed grooves. The antibacterial effect and cytotoxicity may depend on the amount of Ag-NPs embedded on the surface. When the Ag-NPs content was optimal, TC4/Ag MMNC effectively enhance the activity and proliferation ability of BMSCS *in vitro*, with effective antibacterial performance and good biocompatibility. Ag-NPs on the implant surface could promote ROS generation, whereas Cu-NPs could not (van Hengel et al., 2020). The same conclusion goes for (Šulce et al., 2016). They showed that the generated ROS is actually induced by Cu ions released from Cu-NPs.

Optimized surface structure and modifications may not only reduce bacterial adhesion but even attractive to osteoblasts, thereby stimulating osseointegration. The surface topography on nanoparticles coating can regulate cell and bacterial adhesion through different structures. Li et al. (2017) designed hybird ZnO nanorods with an average diameter of 100 nm on to titanium which not only can effectively kill bacteria simultaneously but also enhance the osteoinductivity simultaneously. It is because osteoblasts are approximately an order of magnitude larger than bacteria in size, there is a great difference in the sensitivity of osteoblasts and bacteria to nanorod arrays. This suggest that we can take advantage of the difference in sensitivity of bacteria and osteoblasts to certain simple factor to endow the implant surface with osteogenic and antibacterial abilities. Extracellular pH plays an important role to affect bone formation/resorption and bacterial growth (Tan et al., 2018). Most of bacteria cannot grow well in a micro-environment with pH over 9.0 (Padan et al., 2005). However, alkaline micro-environment can enhance the proliferation and differentiation of osteoblasts and promote bone formation (Arnett, 2008; Tan et al., 2018). Tao et al. (2020) developed a strategy fabricating nanoparticles coating on Ti implants with collagen-modified metal-organic frameworks (MOF)@Levofloxacin (Levo), which displays pH-response, while then inducing alkaline micro-environment. The released OH^−^ can react with H^+^ in bacteria continuously, thereby reducing the ATP level of bacteria. The intracellular ROS level was significantly increased due to alkaline micro-environment and Zn^2+^. Delightfully, the pH-response multifunctional coating not only inhibiting *E. coli* and *S. aureus*, but also performing positive effects on osteogenic activity such as accelerating osteoblast proliferation and differentiation, upregulating osteogenic-relative gene expression and presenting positive ossointegration effect *in vivo* which probably related to the alkaline micro-environment. Tao and co-workers focused on developing biomaterials with immunomodulatory functions in their subsequent studies (Tao et al., 2021). Recently, they prepared a multifunctional hybrid coating by deposition of zeolitic imidazole frameworks-67 (ZIF-67) and osteogenic growth peptide (OGP) was fabricated on titanium dioxide nanotubes (TNT). The enhanced osteogenic capability and immunoreaction of TNT-ZIF-67@OGP *in vitro* and *in vivo* were attributed to the formation of an alkaline micro-environment, the release of OGP as well as anti-inflammatory properties. At the same time, the production of ROS, reduction of ATP level and the coating dissolved under the acidic environment, releasing Co^2+^ and forming an alkaline microenvironment to kill bacteria. In addition, TNTs also have been demonstrated to enhance the expression of osteogenic genes compared with titanium (Tao et al., 2019).

#### 4.2.2 Photosensitive antibacterial surface

Killing bacteria by photocatalyst-induced ROS has proved to be an effective strategy for preventing bacteria-related infections due to the inability to develop bacterial resistance (Wei et al., 2019). The antimicrobial photodynamic therapy attracted more attention due to its higher antibacterial efficiency and safety.

TiO_2_ is commonly used as photocatalytic material, which shows strong photo-oxidation ability when illuminated (Ohtsu et al., 2015). TiO_2_ is used as the main component of the stable titanium oxide layer on the surface of the titanium-based material, which thickness is only a few nanometers. In brief, under the excitation of UV light, the TiO_2_ with properties surface can generate electron-holes pairs and initiate a series of photocatalytic reactions (Chouirfa et al., 2019; Alotaibi et al., 2021). When activated by UV light of sufficient energy, electrons are excited from the valence to the conduction band, leaving holes (Gallardo-Moreno et al., 2010). Photogenerated electrons and holes can diffuse to the surface of TiO_2_ and react with O_2_ and H_2_O to generate ROS, such as O_2_^−^ and OH. Hydroxyl radical can damage DNA, nucleic acids and amino acids, while O_2_^−^ can damage tissues and degrade cell membrane (Sunada et al., 2003; Alotaibi et al., 2021). Hatoko et al. (2019) studied the photocatalytic antibacterial activity of TNS which created by the deposition of Ti *via* TiO_2_ sputtering. On UV-treated TNS surface, the growth of *S. aureus* was obviously inhibited after 6 h. By detecting ROS with DCFH-DA probe, they found that antibacterial effect was caused by oxidative stress. It can be considered that UV irradiation improved the antibacterial properties of TNS and reduced the viability of *S. aureus* on the material surface by producing ROS.

The photocatalytic antibacterial activity is related to the crystal from of the hybrid materials (Foster et al., 2011; Liao et al., 2020). The TiO_2_ oxide film on the surface of Ti generally exists in an amorphous state and does not exhibit photocatalytic activity. However, three crystal structures of TiO_2_ such as anatase, rutile and brookite can be made photocatalytic activity by various oxidation methods (He et al., 2019). Although the light absorption capacity of anatase is lower than that of rutile, the band gap (3.2 eV) of anatase is larger than that of rutile (3.0 eV) (Liao et al., 2020). Therefore, it can be considered that the photocatalytic activity of anatase is preferable to that of rutile. Chen Y. et al. (2019) proposed that the exist of Ti-O-Si bond in TiO_2_@SiO_2_ hybrid materials can effectively inhibit the transformation of TiO_2_ from the anatase into rutile, which was easier for the production of ROS such as hydroxyl radicals and further improvement of the antibacterial performance. The addition of ZnO also obviously promotes the antibacterial effect of the material. Compared with the most common modification of TiO_2_, the band gap energy of ZnO is slightly higher, reaching 3.37 eV (Coleman and Jagadish, 2006). Samples containing ZnO have a higher antibacterial rate than samples without ZnO under photocatalytic conditions (Zhu et al., 2017), and ROS produced by ZnO photocatalysis also have great influence on antibacterial performance, the disruption of membrane can be observed in SEM image.

Unfortunately, the effect of UV irradiation shows a rapid decay, without the continuing antibacterial effect (Monetta and Bellucci, 2014). The depth of UV penetration into the tissue is limited, it is unclear whether it is effective for deep periodontal pockets around implants (Zhang E. et al., 2021), and prolonged exposure is also harmful to tissues. ROS generated upon photostimulation have different diffusion degrees to different types of bacteria, and the ROS generation ability will gradually disappear after the external stimulation turned off (Sperandio et al., 2013). Whether it can be used on the surface of implants needs in-depth study.

#### 4.2.3 Plasma treatment surface

Plasma, a neutral ionized gas considered to be the fourth fundamental state of matter (in addition to solids, liquids, and gases), with particles (photons, electrons, positive and negative ions, atoms, free radicals, and excited or non-excited molecules) in permanent interaction (Monetta and Bellucci, 2014). Therefore, it can be considered that the presence of plasma may have a general oxidative effect on the cell surface layer of microorganisms, inhibiting their growth or metabolic activity. Plasma can be divided into thermal and non-thermal plasma according to the temperature (Chen, 1984). However, the thermal plasma with the temperature range of a 10th of thousands and up to millions of Kelvins, which make it less applicable as surface processes (Bencina et al., 2021).

Reactive oxygen and nitrogen species are the main active components of non-thermal plasma, enabling titanium surface perform reductive potential which oxidize the surrounding material (Lee et al., 2019; Shao et al., 2022). Monetta and Bellucci (2014) proposed that oxygen cold plasma treatment can increase the content of TiO_2_ on the surface of titanium samples and promote the anatase formation. Without UV irradiation, it still shows high antibacterial effect, which related to ROS production. In another study, Lee et al. (2019) treat the surface of Ti by atmospheric plasma jet (APPJ). The plasma-treated Ti surface has a reduction potential, leading to oxidation of the surrounding environment. In addition, TiO_2_ could be modified by ions, such as COOH^−^, OH^−^, NO^−^, N^3−^and O^2-^ after plasma treatment (Lee et al., 2019; Shao et al., 2022). Unfortunately, this type of treatment has only a temporary effect on bacterial adhesion, as the ions remain on the surface only for a limited time (Bencina et al., 2021).

For nanoparticles coatings, the antibacterial activity can be improved by adjusting the relevant process parameters of plasma. Ye et al. (2020) studied the effect of different voltage treatments on the antibacterial effect of Zn-doped TiO_2_ coatings during plasma electrolytic oxidation (PEO) treatment. Compared with the PEO-300 V and PEO-400 V coatings, increasing HO·, O_2_^−^ and H_2_O_2_ content as well as the release of Zn^2+^ generated on the PEO-530 V coating can be attributed to the weaken of Zn-O bonds in amorphism generated by the PEO treatment. The H_2_O_2_ and Zn^2+^ produced on the coating induced intracellular ROS production, which further aggravates plasma membrane damaged and contents released, leading to bacterial death. Not only voltage, but also the time of treatment process can affect the antibacterial effect. In the Ag-NPs@Ti prepared by Plasma immersion ion implantation (PIII) technology (Wang et al., 2017), as the ion implantation time from 1 to 3 h, the corrosion current increased which more easily activate the micro-galvanic couples and triggering electron transfer (Cao et al., 2011). As mentioned above, ROS was generated by micro-galvanic couples and electron transport in this study.

## 5 Conclusion and perspectives

In summary, ROS may act as an “intermediate messenger” in the communication between the implant and the host environment. A variety of ways can improve the ROS response of implants, the host’s response to implants can be adjusted to accelerate bone integration. Besides, various materials for implant fabrication also show excellent antibacterial effect *in vitro* experiments by controlling oxidative stress. In this paper, the research progress of antibacterial function and improving biocompatibility of titanium alloys with ROS response were reviewed, and the related mechanisms in recent years were summarized. We introduced that oxidative stress can be affected by the nature of the material itself, such as the size of degradation products, wettability, mechanical properties of the material. In addition, some inappropriate factors also lead to the increase of oxidative stress products and affect some basic properties of the material through electrochemical pathways, such as corrosion resistance and biocompatibility. Importantly, the implant material can utilize the generation of ROS *in vitro* to promote cell proliferation and stimulate M2 polarization of macrophages, as well as exert an antibacterial effect, prevent peri-implant inflammation, and allow the wound to enter a healing stage more quickly.

Although the research for ROS made great achievements in the fields of molecular biology and materials science, many problems remain to be studied. Most of the current articles focus on the synergistic antibacterial effect of multiple factors, and lack of studies concentrate on the antibacterial mechanism of ROS alone. Besides, most of the experiments in this part were conducted *in vitro* in which the selection of the test strains is too single, and some common pathogenic bacteria in the oral cavity had not been studied yet. Therefore, deeper research *in vivo* is required, especially to evaluate its impact on long-term implantation, which determines whether it can be applied to clinical work. Finally, the long-term cytotoxicity of active elements and the wear of materials during implantation should also be widely discussed in the study of ROS regulation by doping active elements or surface modification of the implant.

ROS plays a “double-edged sword” role in implantation healing process which directly influence the success rate of implantation surgery. Reasonable control of redox balance may be the key to prevent implant loosening and degradation and to achieve long-term implant stability. In addition, the smart use of ROS can help us to develop new implant materials with both antibacterial properties and good biocompatibility. To this end, designing good biomaterials with ROS responsiveness is a promising strategy to improve their *in vivo* outcomes.
